# Prolonged Cholestatic Hepatitis A in an Unvaccinated International Traveler

**DOI:** 10.7759/cureus.101110

**Published:** 2026-01-08

**Authors:** Erika Yuki M Bomfim, João Vitor M Viana, Gabriella Cecília Vanin, Mariana S Kajita, Marcos V da Silva

**Affiliations:** 1 Infectious Diseases, Institute of Infectious Diseases Emilio Ribas, São Paulo, BRA

**Keywords:** cholestasis, hepatitis a, travelers’ health, travel medicine, vaccination

## Abstract

Hepatitis A is a viral infection that is generally self-limited, although atypical, prolonged forms and fatal outcomes may occur. This case report describes a 33-year-old Brazilian man, previously healthy and unvaccinated, who developed prolonged cholestatic hepatitis A acquired during international travel. He had traveled through areas with precarious sanitation and consumed untreated water in a rural region of Zambia, Africa. Initial symptoms were nonspecific and progressed to jaundice, dark urine, and acholic stools. The diagnosis of acute hepatitis A was confirmed serologically, with markedly elevated transaminases and predominantly direct hyperbilirubinemia. Despite initial clinical improvement, he persisted with jaundice and diarrhea for more than two months, with continued fecal shedding of hepatitis A virus (HAV) RNA. Treatment included symptomatic medications and ursodeoxycholic acid due to cholestasis, with good clinical resolution after four months. This report aims to highlight the prolonged course of hepatitis A in adults, distinct from the typically faster resolution observed in children, and to reinforce the importance of vaccination among the general population and in travel medicine counseling, especially for adults from countries with low endemicity for hepatitis A.

## Introduction

Hepatitis A is a viral infection caused by an enteric picornavirus, whose genome consists of a single-stranded RNA molecule. Transmission occurs primarily via the fecal-oral route, through ingestion of contaminated water or food, or via interpersonal contact, including oral-anal sexual practices [1-3]. The incubation period ranges from 15 to 50 days, with a mean of four weeks [4,5]. Clinical disease generally lasts one to two weeks; however, 10% to 15% of cases may show atypical prolonged symptoms or relapse within six to nine months [3,6]. One of these atypical manifestations is the cholestatic presentation, characterized by prolonged jaundice, pruritus, acholic stools, and choluria lasting around 12 weeks. Fecal shedding of hepatitis A virus (HAV) begins approximately one to two weeks before symptom onset and typically lasts two months, although it may persist for up to six months in children, posing a risk of transmission even in the absence of clinical manifestations [3,5].

Hepatitis A presents with a wide spectrum of clinical manifestations, ranging from mild, self-limited disease to severe acute liver failure and death, events more common in adults due to immunomediated mechanisms (innate and adaptive) rather than direct viral cytopathic effects [3,4]. The onset is abrupt and often marked by nonspecific symptoms such as fever (18-75%), malaise (52-91%), nausea and vomiting (26-87%), and abdominal discomfort (37-65%) [3,4]. After a few days, typically within one week, the icteric phase begins, characterized by jaundice (40-80%), dark urine (28-94%), and hepatomegaly (78%), along with significant laboratory abnormalities including elevated transaminases and increased bilirubin, gamma-glutamyl transferase, and alkaline phosphatase levels [3,4,6]. Less common symptoms include pruritus, diarrhea, arthralgia, and rash [4,6,7]. Approximately 80% of patients achieve complete clinical and laboratory recovery within three months [3].

The disease has a universal distribution, with higher incidence in areas lacking basic sanitation [1,2]. Even in low-endemicity regions, outbreaks remain a public health concern and are strongly associated with a lack of immunization [8]. In Brazil, hepatitis A vaccination was included in the National Immunization Program (PNI) for children up to five years old in 2014 [9,10], contributing, along with improvements in sanitation, to a reduction in disease incidence [11-13]. Nonetheless, unexposed or unvaccinated individuals remain susceptible, particularly young adults from middle- and upper-class backgrounds and international travelers [8,13,14].

This report describes a case of cholestatic hepatitis A with a prolonged course in an international traveler without prior immunity.

This article was previously presented as an academic poster at the 14th São Paulo Congress of Infectious Diseases on August 29, 2024.

## Case presentation

A 33-year-old Brazilian male, previously healthy, sought medical attention due to jaundice and diarrhea that had begun two months earlier. He had been abroad for nine months on a tourism and volunteer trip. His itinerary included India, Vietnam, Thailand, South Korea, Cambodia, Indonesia, Singapore, Zambia, Zimbabwe, Botswana, and South Africa. He stayed in Zambia, where the symptoms started, for 45 days performing volunteer work in rural villages, where there was no electricity or sanitation system; waste disposal occurred in dry latrines. The water supply was untreated river water, and food was locally produced.

While in Africa, he sought medical care in Johannesburg, South Africa, due to diffuse myalgia, headache, colicky abdominal pain, nausea, vomiting, and high fever (39°C). Laboratory tests for malaria and dengue were negative, and he was prescribed symptomatic treatment, with fever improvement.

He continued traveling, and 16 days after symptom onset, while in Thailand, he developed jaundice, dark urine, and acholic stools. At a hospital in Bangkok, laboratory tests showed elevated ALT (1988 U/L) and direct bilirubin (7.2 mg/dL), as shown in Table 1. Abdominal CT revealed hepatomegaly (18.2 cm measured at the right mid-clavicular line), slight parenchymal attenuation, and periportal hypodensity. Serologies for hepatitis A, B, and C were performed, with reactive anti-HAV IgM. He was discharged after three days of hospitalization with improved transaminases. Fifteen days later, he returned to the same hospital because of persistent jaundice and diarrhea. Laboratory tests showed near-normal transaminases (aspartate aminotransferase (AST) 71 U/L, alanine aminotransferase (ALT) 117 U/L) but markedly elevated bilirubin (total bilirubin 20.5 mg/dL). Stool tests for enteroparasites were negative. Due to the persistence of symptoms for more than three weeks, he underwent further evaluation, including repeat laboratory tests (total bilirubin 21.5 mg/dL, direct bilirubin 14.3 mg/dL, AST 109 U/L, ALT 175 U/L, alkaline phosphatase 187 U/L) and abdominal ultrasound, which showed mild hepatomegaly with periportal edema, thickened gallbladder wall, and mild splenomegaly. Treatment with vitamin K, ursodeoxycholic acid, and Silybum marianum (herbal medicine) was prescribed, considering the diagnosis of cholestatic hepatitis A.

**Table 1:** Chronological laboratory liver evaluation consistent with cholestatic hepatitis due to persistent direct hyperbilirubinemia despite improving transaminases. Unfortunately some laboratorial data is missing. TB: total bilirubin, DB: direct bilirubin, ALT: alanine aminotransferase, AST: aspartate aminotransferase, ALP: alkaline phosphatase, GGT: gamma-glutamyl transferase.

Exams	Thailand Day 16	Thailand Day 31	Thailand Day 52	Brazil Day 60	Brazil Day 120	Reference Values
TB	9.4	20.5	21.5	19.1	2.3	0.2-1.3 mg/dL
DB	7.2	13.6	14.3	16.2	2.2	0.1-0.5 mg/dL
ALT	1988	117	175	226	47	<50 U/L
AST	409	71	109	129	28	<59 U/L
ALP	123	139	187	193	-	38-126 U/L
GGT	-	-	-	55	-	15-73 U/L

**Figure 1:**
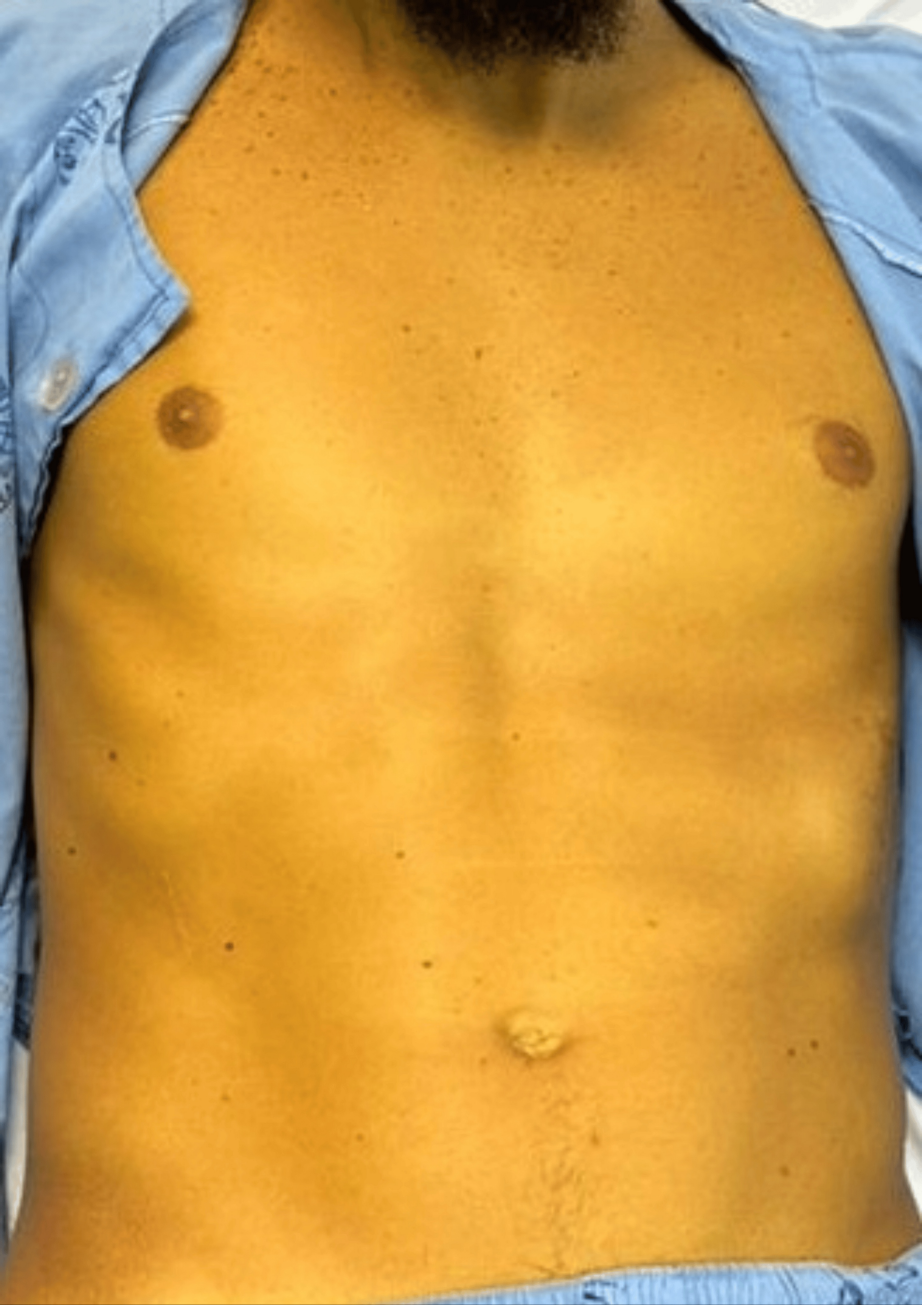
Clinical image showing diffuse, intense jaundice involving the trunk and upper extremities, consistent with the cholestatic phase of acute hepatitis A due to prolonged direct hyperbilirubinemia. The photo was taken 60 days after the onset of the symptoms.

The patient returned to Brazil two months after symptom onset. Twenty-five days later, he again experienced malaise, myalgia, headache, abdominal pain, nausea, vomiting, diarrhea, and fever around 39°C, with persistent jaundice, pruritus, and diarrhea. He sought medical evaluation because of the persistence of diffuse and intense jaundice for several weeks despite improvement in transaminase levels (Figure 1).

Serologies for viral hepatitis, BioFire® FilmArray® Gastrointestinal (GI) Panel testing, and PCR testing for viral hepatitis A and E (HEV) in blood and stool were performed; the results are shown in Table 2. 

**Table 2:** Results of serological and molecular assays performed for etiological elucidation, demonstrating reactive anti-HAV markers, persistent fecal HAV RNA detection, and exclusion of other viral, bacterial, and parasitic gastrointestinal pathogens. HAV: hepatitis A virus; IgM: immunoglobulin M; HBsAg: hepatitis B surface antigen; Anti-HBc: antibody against hepatitis B virus core antigen; Anti-HBs: antibodies against hepatitis B surface antigen; Anti-HCV: antibodies against hepatitis C virus; PCR: polymerase chain reaction; HEV: hepatitis E virus.

Test	Components	Specimen	Result	Reference Values
HAV - total antibodies	-	Blood	Reactive	Non-reactive
HAV - IgM	-	Blood	Reactive	Non-reactive
HBsAg	-	Blood	Non-reactive	Non-reactive
Anti-HBc total	-	Blood	Non-reactive	Non-reactive
Anti-HBs	-	Blood	0,00 mIU/mL	Non-reactive < 8mIU/mL; Indeterminate: 8-12 mIU/mL; Reactive > 12 mIU/mL
Anti-HCV total	-	Blood	Non-reactive	Non-reactive
PCR HAV	-	Blood	Not detected	Not detected
PCR HAV	-	Stool	Detected	Not detected
PCR HEV	-	Blood	Not detected	Not detected
PCR HEV	-	Stool	Not detected	Not detected
BioFire^® ^FilmArray^® ^Gastrointestinal (GI) Panel (bioMérieux, Lyon, France)	Campylobacter	Stool	Not detected	Not detected
	*Clostridioides difficile* toxins A/B	Stool	Not detected	Not detected
	Plesiomonas shigelloides	Stool	Not detected	Not detected
	*Salmonella *spp.	Stool	Not detected	Not detected
	*Vibrio *spp.	Stool	Not detected	Not detected
	Vibrio cholerae	Stool	Not detected	Not detected
	Yersinia enterocolitica	Stool	Not detected	Not detected
	Enteroaggregative *E. coli* (EAEC)	Stool	Not detected	Not detected
	Enteropathogenic *E. coli* (EPEC)	Stool	Not detected	Not detected
	Enterotoxigenic *E. coli* (ETEC) stx1/stx2	Stool	Not detected	Not detected
	*Cryptosporidium *spp.	Stool	Not detected	Not detected
	Cyclospora cayetanensis	Stool	Not detected	Not detected
	Entamoeba histolytica	Stool	Not detected	Not detected
	Giardia lamblia	Stool	Not detected	Not detected
	Adenovirus F 40/41	Stool	Not detected	Not detected
	Astrovirus	Stool	Not detected	Not detected
	Norovirus GI/GII	Stool	Not detected	Not detected
	Rotavirus A	Stool	Not detected	Not detected
	Sapovirus	Stool	Not detected	Not detected

The patient was instructed to observe strict sanitary measures, since he was excreting the virus into the environment, posing a risk of spreading the disease. He showed gradual laboratory improvement and achieved full clinical recovery four months after symptom onset.

## Discussion

Hepatitis A is typically a self-limited viral infection, although atypical and prolonged clinical presentations, such as in this case, may occur [6,14]. The cholestatic variant, characterized by persistent jaundice, intense pruritus, elevated bilirubin, especially direct, dark urine, acholic stools, and mild elevations of canalicular enzymes, occurs in about 5% of cases and may last for weeks to months [3,6,14-16]. In this case, the laboratory pattern corresponded with a cholestatic presentation due to persistent hyperbilirubinemia despite improving transaminases. While not associated with a worse prognosis, this form requires prolonged follow-up and entails greater morbidity [3]. This clinical course is compatible with the atypical manifestations of HAV infection reported in the literature, which could extend up to 40 weeks [3,6].

According to the literature, our patient had clear epidemiological risk factors for HAV infection, including lack of prior vaccination or disease and exposure to environments with poor sanitation and high endemicity [1,3,5]. During his stay in rural Zambia, he consumed untreated river water and food prepared under precarious hygiene conditions [1-3].

Lack of HAV immunization is a vulnerability factor in countries undergoing epidemiological transition, such as Brazil, where improved sanitation has reduced early childhood exposure, leaving unvaccinated adults susceptible [8,11,12]. Since 2014, hepatitis A vaccination has been part of the National Immunization Program for children up to five years of age, but it is not routinely offered to adults [9,17]. In Brazil’s public health system, adult vaccination is reserved for specific groups, including immunocompromised individuals, users of HIV pre-exposure prophylaxis, and persons with chronic liver disease [18,19]. Among international travelers, HAV remains an important cause of acute illness and is one of the main vaccine-preventable infections in this population [13,17].

In this case, the persistence of jaundice and diarrhea despite normalization of transaminases reflected a prolonged cholestatic course, prompting investigation for other etiologies. Exclusion of alternative infectious or hepatic causes, combined with the detection of HAV RNA (qualitative analysis by reverse transcription and polymerase chain reaction amplification of the VP1/P2a region of the hepatitis A virus genome) in stool for more than two months, supported the diagnosis. A study [4] shows that HAV RNA may remain detectable in feces for prolonged periods even after resolution of viremia, due to persistence of the virus in the liver, which sustains fecal excretion.

Management of such cases consists primarily of supportive care; ursodeoxycholic acid may be used, as in the present case, although its efficacy is not fully established in clinical trials [4]. It is commonly used in practice to alleviate symptoms caused by cholestasis [3,16]. The herbal compound Silybum marianum has empirical use but limited scientific evidence. The use of this medicine did not result in subsequent biochemical or clinical improvement in our patient, as observed in Table 1.

This case underscores the importance of preventive strategies, including vaccination during pre-travel consultations, and awareness of atypical clinical manifestations of hepatitis A, especially in the context of global human mobility and the eco-epidemiology of infectious diseases.

## Conclusions

Hepatitis A may present with a range of clinical forms, from mild disease to fulminant hepatitis with hepatic necrosis. The incorporation of HAV vaccination into Brazil’s National Immunization Program in 2014, along with improvements in sanitation, has reduced the incidence of this infection. However, unvaccinated adults, particularly young adults from middle- and upper-income groups and individuals traveling domestically or internationally, remain at risk for clinically significant disease that may require hospitalization or lead to death. This case highlights the importance of epidemiological surveillance for HAV and preventive strategies such as pre-travel vaccination for adults.

## References

[1] Cao G, Jing W, Liu J, Liu M (2021). The global trends and regional differences in incidence and mortality of hepatitis A from 1990 to 2019 and implications for its prevention. Hepatol Int.

[2] Kirk MD, Pires SM, Black RE (2015). World Health Organization estimates of the global and regional disease burden of 22 foodborne bacterial, protozoal, and viral diseases, 2010: a data synthesis. PLoS Med.

[3] Ciocca M (2000). Clinical course and consequences of hepatitis A infection. Vaccine.

[4] Shin EC, Jeong SH (2018). Natural history, clinical manifestations, and pathogenesis of hepatitis A. Cold Spring Harb Perspect Med.

[5] Foster MA, Haber P, Nelson NP (2024). Hepatitis A. CDC Pink Book.

[6] Schiff ER (1992). Atypical clinical manifestations of hepatitis A. Vaccine.

[7] Kim JH, Yeon JE, Baik SK (2013). Genotypic shift of the hepatitis A virus and its clinical impact on acute hepatitis A in Korea: a nationwide multicenter study. Scand J Infect Dis.

[8] Jacobsen KH (2018). Globalization and the changing epidemiology of hepatitis A virus. Cold Spring Harb Perspect Med.

[9] De Soárez PC, Sartori AM, Santos A, Itria A, Novaes HM, Martelli CM (2012). Contributions from the systematic review of economic evaluations: the case of childhood hepatitis A vaccination in Brazil. Cad Saude Publica.

[10] Brasil. Ministério da Saúde. Secretaria de Vigilância em Saúde (2025). Brasil. Ministério da Saúde. Secretaria de Vigilância em Saúde: Informe técnico da introdução da vacina adsorvida hepatite A (inativada). Ministério da Saúde, Brasília, Brazil. Brasília: Ministério da Saúde.

[11] Ximenes RA, Martelli CM, Amaku M (2014). Modelling the force of infection for hepatitis A in an urban population-based survey: a comparison of transmission patterns in Brazilian macro-regions. PLoS One.

[12] Van Effelterre T, Guignard A, Marano C, Rojas R, Jacobsen KH (2017). Modeling the hepatitis A epidemiological transition in Brazil and Mexico. Hum Vaccin Immunother.

[13] Balogun O, Brown A, Angelo KM (2022). Acute hepatitis A in international travellers: a GeoSentinel analysis, 2008-2020. J Travel Med.

[14] Jung YM, Park SJ, Kim JS (2010). Atypical manifestations of hepatitis A infection: a prospective, multicenter study in Korea. J Med Virol.

[15] Migueres M, Lhomme S, Izopet J (2021). Hepatitis A: epidemiology, high-risk groups, prevention and research on antiviral treatment. Viruses.

[16] Brasil. Ministério da Saúde. Secretaria de Vigilância em Saúde e Ambiente. Departamento de Imunizações e Doenças Imunopreveníveis (2023). Departamento de Imunizações e Doenças Imunopreveníveis. Manual dos Centros de Referência para Imunobiológicos Especiais. 6. Manual dos Centros de Referência para Imunobiológicos Especiais, 6th Edition.

[17] Brasil. Ministério da Saúde (2025). Brasil. Ministério da Saúde. Secretaria de Vigilância em Saúde e Ambiente. Programa Nacional de Imunizações. Secretaria de Vigilância em Saúde e Ambiente. Programa Nacional de Imunizações [access.

[18] Prahraj D, Anand AC (2021). Tropical liver diseases: an overview. Clin Liver Dis (Hoboken).

[19] El-Kamary SS, Shardell MD, Abdel-Hamid M (2009). A randomized controlled trial to assess the safety and efficacy of silymarin on symptoms, signs and biomarkers of acute hepatitis. Phytomedicine.

